# Commensal microflora induce host defense and decrease bacterial translocation in burn mice through toll-like receptor 4

**DOI:** 10.1186/1423-0127-17-48

**Published:** 2010-06-12

**Authors:** Lee-Wei Chen, Wei-Jung Chang, Pei-Hsuan Chen, Ching-Mei Hsu

**Affiliations:** 1 Institute of Emergency and Critical Care Medicine, National Yang-Ming University, Taipei, Taiwan; 2 Department of Surgery, Kaohsiung Veterans General Hospital, 386, Ta-chung 1st Road, Kaohsiung, Taiwan; 3 Department of Biological Sciences, National Sun Yat-Sen University, 70 Lien-Hai Road, Kaohsiung 804, Taiwan

## Abstract

**Background:**

Major burn is associated with decreased gut barrier function and increased bacterial translocation (BT). This study is to investigate whether commensal microflora induce host defense and decrease BT in burn mice.

**Methods:**

First, we treated Wild type (WT) mice with antibiotics in drinking water for 4 weeks to deplete gut commensal microflora. At week 3, drinking water was supplemented with lipopolysaccharide (LPS); a ligand for TLR4, to trigger TLRs in gut. The intestinal permeability, glutathione level, NF-κB DNA-binding activity, TLR4 expression of intestinal mucosa, BT to mesenteric lymph nodes (MLNs), and bacterial killing activity of peritoneal cells were measured after thermal injury. Second, lung of animals were harvested for MPO activity and TNFα mRNA expression assay. Third, WT animals were treated with oral antibiotics with or without LPS supplement after burn. At 48 hr after burn, TLR4 expression of intestinal mucosa and bacterial killing activity of cells were examined. Finally, bacterial killing activity and BT to MLNs after thermal injury in C3H/HeJ (TLR4 mutant) mice were measured.

**Results:**

Burn induced BT to MLNs in WT mice. Commensal depletion decreased TLR4 expression as well as NF-κB activation of intestine, myeloperoxidase (MPO) activity as well as TNFα expression of lung, and bacterial killing activity of peritoneal cells. Oral LPS supplement markedly reduced 81% of burn-induced BT and increased TLR4 expression, MPO activity of lung, as well as bacterial killing activity of peritoneal cells. LPS supplement did not change BT or bacterial killing activity in C3H/HeJ mice.

**Conclusions:**

Collectively, commensal microflora induce TLR4 expression of intestine and bacterial killing activity of inflammatory cells in burn. TLR4 ligand increases bacterial killing activity and decreases burn-induced BT. Taken together with the abolition of LPS effect in TLR4 mutant mice, we conclude that commensal microflora induce host defense and decrease bacterial translocation in burn mice through toll-like receptor 4.

## Background

The human intestines are colonized by trillions of microorganisms, including hundreds of different species of bacteria and viruses [1]. These microbes, collectively referred to as the commensal microflora, have an important role in human nutrition and health, by promoting nutrient supply, preventing pathogen colonization and shaping and maintaining normal mucosal immunity [2]. Major burn in humans and experimental animals is associated with a decrease in immune functions dependent upon T cells, the principal cells involved in initiating adaptive immune responses [3]. It is well accepted that clinical or experimental burn injury disrupts gut barrier function, allows dissemination of bacteria from the intestinal tract and facilitates the bacterial translocation (BT) to MLN, liver, and spleen [4,5]. The magnitude of BT is proportional to the severity of the burn injury [6]. Previously, burn surgeons believed that feeding should not be started during the first 48 to 72 hours after burn or after injury because of theory that there was an obligatory pan-intestinal ileus. Today, there is overwhelming evidence that provision of enteral nutrients shortly after injury alters gut flora and protects the immuno-compromise, stressed, or thermally injured patients through unknown mechanism [7,8]. Defining the relationship between commensal microflora and gut barrier function is warranted to characterize the beneficial effect of early enteral feeding in burn patients.

The innate immune system detects the invasion of microorganism through toll-like receptors (TLRs), which recognize microbial components and trigger inflammatory responses. TLRs comprise a family of pattern-recognition receptors that detect conserved molecular products of microorganisms [9,10]. TLRs function as sensors of microbial infection and are critical for the initiation of inflammatory and immune defense responses. TLR2 and TLR4, have been shown to be essential for the recognition of distinct bacterial cell wall components. TLR2 discriminates peptidoglycan (PGN), lipoprotein, lipoarabinomannan (LAM) and zymosan, whereas TLR4 recognizes lipopolysaccharide (LPS), lipoteichoic acid (LTA) and Taxol [11]. A major downstream effect of TLR signaling is the activation of the transcription factor NF-κB, which is required for expression of many genes related to innate immunity and inflammation [12]. Previous paper indicated that toll-like receptors played crucial roles in the maintenance of intestinal epithelial homeostasis [13]. The bacterial ligands recognized by TLRs are not unique to pathogens, but rather are shared by entire classes of bacteria, and are produced therefore by commensal microorganisms as well [13]. However, it is still not decided whether oral supplement TLR4 ligand could enhance host defense in burn or not.

The role of commensal microflora in maintaining innate immunity after thermal injury has not been well characterized. Also, effect of TLR4 ligand on the bacterial killing activity of inflammatory cells after burn has not been examined. We hypothesized that commensal microflora in gut maintained host defense against bacterial challenge and TLR4 ligand decreased burn-induced BT in burn. Using a commensal depletion model in wild-type and TLR4 mutant mice, we demonstrated that both commensal microflora and oral TLR4 ligand enhanced the gut barrier function in burn through the increase of toll-like receptor 4 expression. In the future, enteral feeding of TLRs ligand could be a feasible way to increase host defense and gut barrier function in burn or major trauma patients.

## Methods

### Animals

Specific pathogen-free male C3H/HeN (wild-type, WT), weighing between 20 and 25 g were obtained from the National Laboratory Breeding and Research Center (NLBRC, Taipei, Taiwan). C3H/HeJ (TLR4 mutant) mice were purchased from The Jackson Laboratory (Bar Harbor, ME). C3H/HeJ mice have been demonstrated to have a missense mutation in the third exon of TLR4, yielding a nonfunctional TLR4 [14]. All animal procedures were in compliance with regulations on animal used for experimental and other scientific purposes approved by the National Sun Yat-Sen University Animal Experiments Committee.

### Experimental design

#### Experiment 1

To evaluate the role of commensal microflora on thermal injury-induced intestinal barrier dysfunction, WT mice were fed with vehicle or oral antibiotics for 4 wks to deplete the intestinal commensals with or without LPS supplements in drinking water (10 μg/μl) at week 3. Wild type (WT) mice were randomly divided into four sham groups (control, LPS, antibiotics, antibiotics + LPS) (n = 6) and four burn groups (control, LPS, antibiotics, antibiotics + LPS) (n = 6 in each). The sham group was subjected to sham treatment and the burn groups were subjected to a 30 - 35% total body surface area (TBSA) burn injury. All animals received sterile saline (50 ml/kg i.p.) for fluid resuscitation right after burn or sham treatment. At 24 hr after burn, mesenteric lymph nodes were harvested for bacterial translocation assay. Also, the distribution of fluorescein isothiocyanate-dextran (FITC-dextran) across the lumen of small intestine in animals under anesthesia (ketamine and xylazine) was measured at 24 hr after injury to assess the intestinal permeability. In another experiment, the GSH level of the intestinal mucosa in animals with the same quantity and treatment was measured to assess the peroxidation produced after injury. Mid-ileum tissues were harvested for TLR4 immunohistochemical studies. In another experiment, the intestinal mucosa was harvested for NF-κB DNA-binding activity, TLR4 mRNA and protein expression assay at 8 hr after burn injury.

#### Experiment 2

To evaluate the role of commensal microflora on thermal injury-induced neutrophil deposition and cytokines expression in lung, WT mice were randomly divided into four sham groups (n = 6) and four burn groups (n = 6 in each) as experiment 1. The animals were sacrificed at 8 hr after burn, and lung tissue was harvested for MPO activity. In another experiment, the lung tissue was harvested for the assay of TLR2, TLR4, and TNFα mRNA expression at 8 hr after injury.

#### Experiment 3

Most major burn patients suffered from ileus and received combined antibiotics treatment to prevent sepsis [15,16]. To evaluate the effect of antibiotics treatment with or without LPS supplement in the thermal injury-induced bacterial translocation, WT mice were randomly divided into one sham burn groups (n = 6) and three burn groups (burn, antibiotics, antibiotics + LPS) (n = 6 in each). The sham group was subjected to sham treatment and oral saline feeding. The burn group was subjected to burn treatment and oral saline feeding. The antibiotics group was subjected to oral antibiotics administration after burn. The antibiotics + LPS group was subjected to LPS supplements (10 μg/μl) in oral antibiotics administration after burn. At 48 hr after burn or sham burn, peritoneal cells as well as bone marrow cells were harvested for bacterial killing activity, mesenteric lymph nodes were harvested for bacterial translocation, and intestinal mucosa was harvested for TLR4 mRNA assay.

#### Experiment 4

C3H/HeJ mice were randomly divided into three sham groups (n = 6) and three burn groups (n = 6 in each) as experiment 1. At 8 hr after thermal injury, lung was harvested for TLR4 mRNA assay, intestinal mucosa was harvested for TLR4 mRNA assay, and peritoneal cells were harvested from the abdominal cavity for bacterial killing activity and TLR4 as well as TNFα mRNA expression assay.

### Thermal Injury

The thermal injury procedures were modified from those described by Walker *et al *[14]. Briefly, animals were anesthetized intraperitoneally with ketamine (80 mg/kg) and xylazine (10 mg/kg), and a marked area of the shaved dorsal skin was exposed from a wooden template and immersed in 95°C water for 10 sec. This procedure produced a 30 - 35% TBSA burn of the mice. Total body surface area was calculated using murine-specific data [17] and average 40 to 48 cm^2 ^for mice of the weight used. The burn injury caused 8% mortality within the first 4 hrs after burn. Nonsurviving animals were excluded from the subsequent study. The sham control animals were anesthetized, shaved and maintained in identical settings except that room temperature water was used for immersion.

### Depletion of gut commensal microflora and reconstitution of commensal-depleted animals with TLR ligands

Commensal bacterial products have been known to engage TLRs and confer protection against dextran sulfate sodium (DSS)-induced intestinal epithelial injury [13]. Animals were provided ampicillin (A; 1 g/L; Sigma), vancomycin (V; 500 mg/L; Abott Labs), neomycin sulfate (N: 1 g/L; Pharmacia/Upjohn), and metronidazole (M; 1 g/L; Sidmack Labs) in drinking water for four weeks. Previously, a four-week oral administration of vancomycin, neomycin, metronidazole, and ampicillin with the same dose described above in mice has been proved to deplete all detectable commensals [13]. Previously, this oral antibiotics protocol has no significant effect on nutrition and systemic effect [13,18]. To those animals receiving LPS, drinking water was supplemented with 10 μg/μl of purified E. coli 026:B6 LPS (Sigma) at week 3 and continued in drinking water for the duration of sham treatment or thermal injury. LPS, a membrane constituent of gram-negative bacteria, was the best-studied TLR ligand and was recognized by TLR4 and MD-2, a molecule associated with the extracellular domain of TLR4 [19].

### Quantification of intestinal permeability

The assay of intestinal permeability was modified from the method described by Otamiri *et al*. [20]. A 5-cm segment of the jejunum and proximal ileum was dissected with the beginning at 5 cm distal to the ligament of Treitz with well protected superior mesenteric vessels. The bilateral end of the isolated intestine was clamped with rubber bands to prevent the leakage of FITC-dextran. 200 μl of 0.1 M phosphate buffer saline (pH 7.2) containing 25 mg of FITC-dextran (MW 4,400, Sigma) was injected into the lumen. After 30 min, blood sample (100 μl) was taken by a puncture of the portal vein and immediately diluted with 1.9 ml of 50 mM Tris (pH 10.3) containing 150 mM NaCl. The diluted plasma was centrifuged at 4°C, 3,000 g for 7 min and the supernatant was analyzed for FITC-dextran concentration with a fluorescence spectrophotometer (Hitachi, F-2000) at the excitation wavelength of 480 nm and the emission wavelength of 520 nm. Standard curves for calculating the FITC-dextran concentration in the samples were obtained by diluting various amounts of FITC-dextran in a pool of mice plasma, then diluted and centrifuged in the same manner as the samples before measurement.

### Determination of glutathione (GSH) level

The intestinal mucosa glutathione level was quantitated by the fluorescence probe o-phthalaldeyde (sigma) which can react with GSH and has high quantum yield. Mix 1.89ml of 50 mM potassium phosphate buffer (pH8.0) with 10ul of supernatant obtained and add 100 μl of 1mg/ml o-phthalaldeyhyde (freshly prepared in absolute methanol). The samples were incubated at room temperature for 15 min and fluorescence was measured at an excitation wavelength of 350 nm and an emission wavelength of 420 nm. The data was expressed as GSH content (mM).

### Bacterial translocation to MLN

The collected mesenteric lymph nodes were weighed and homogenized in 500 μl of sterile saline. Aliquots of the homogenate from each tissue were plated onto TSB (Tryptic Soy Broth) agar plates (DIFCO, Detroit, Michigan, USA). The plates were examined after aerobic incubation at 37°C for 24 hr to determine whether commensal depletion with or without LPS altered burn-induced translocation. Representative colonies were expressed as colony forming unit per gram of organ tissue (CFU/g tissue).

### Determination of lung myeloperoxidase activity

Lung content of myeloperoxidase (MPO) was determined to assess the degree of pulmonary neutrophil infiltration [21]. Mice were anesthetized and the thorax was opened with median sternotomy. The bilateral lungs and heart were harvested together and the pulmonary vasculature was cleared of blood by gentle injection of 10 ml sterile saline into the right ventricle. The lungs were then blotted dry of surface blood and weighed.

Lung tissues was placed in 50 mM potassium phosphate buffer (pH 6.0) with 0.5% hexadecyltrimethylammonium bromide and homogenized. The homogenate was sonicated on ice and centrifuged for 30 min at 3,000 *g*, 4°C. An aliquot (0.1 ml) of supernatant was added to 2.9 ml of 50 mM potassium phosphate buffer (pH 6.0) containing 0.167 mg/ml of *O*-dianisidine and 0.0005% hydrogen peroxide [22]. The rate of change in absorbance at 460 nm was measured over 3 min. One unit of MPO activity was defined as the amount of enzyme that reduces 1 μmole of peroxide per min and the data were expressed as units per gram of lung tissue (Units/g tissue).

### **Polymerase chain reaction (PCR) and quantification of PCR product***s*

Total RNA was isolated from cells using TRIZOL reagent (Invitrogen, Life Technologies) as described previously [23]. Reverse transcription-generated cDNA encoding TLR2, TLR4, and TNFα genes were amplified using PCR. Sets of primers were designed according to those genes documented in GenBank. The sequences are 5'-AGTGGGTCAAGGAACAGAAGCA-3' (sense) and 5'-CTTTACCAGCTCATTTCTCACC-3' (antisense) for TLR4, 5'-TCTGGGCAGTCTTGAACATTT-3' (sense) and 5'-AGAGTCAGGTGATGGATGTCG-3' (antisense) for TLR2, 5'-CAGCCTCTTCTCATTCCTGCTTGTG-3' (sense) and 5'-CTGGAAGACTCCTCCCAGGTATAT-3' (antisense) for TNFα, and 5' GTGGGCCGCTCTAGGCACCA3' (sense) and 5' CGGTTGGCCTTAGGGTTCAG3' (antisense) for β-actin gene as a control.

### Bacterial killing activity of peritoneal cells and bone marrow cells

The peritoneal cavity was washed with 5 ml PBS containing 0.1% BSA and 10 mM EDTA. The peritoneal cells were collected and resuspended in HBSS as 10^6 ^cells/ml. Bone marrow cells were harvested from bilateral femoral and tibial bone marrow. Red cells depletion was performed using erythrolysis. After 5 min of preincubation, the cell suspension was incubated with *E. coli *(10^8^/ml) at 37°C for 1 h with shaking. The cells were removed as the pellet after centrifugation at 200 × g for 10 min, and *E. coli *number in the supernatant was counted [24,25].

### Western immunoblots

Protein levels of TLR4 of intestinal mucosa were measured by Western immunoblotting. Homogenized samples (50 μg of protein each) were subjected to 12.5% SDS-PAGE under reducing conditions. Proteins were transferred onto PVDF membranes (Millipore) by using a Semi-Dry Electrophoretic system (Bio-Rad). The TLR4 was identified by rabbit monoclonal antibody (Cell Signaling Technology, Inc.). The membranes were incubated with the secondary antibody (Biotinylated anti-rabbit IgG) (Perkin-Elmer Life Science, Boston, USA) for 1 hr at room temperature. Blots were developed by the ECL Western blotting detection reagents (Perkin-Elmer).

### TLR4 immunohistochemistry

The following antibodies were used for immunohistochemical stains: Rabbit monoclonal immunoglobulin G to TLR4 (Cell Signaling Technology, Inc.), biotinylated secondary antibodies and peroxidase-conjugated streptavidin (Dako). The TLR4 antibody with a 1:300 dilution was used. Sections of the paraffin fixed mice lung (4 μm thickness) were deparaffinized with xylene and graded ethanol. For antigen retrieval in the TLR4 staining, sections were soaked in a citrate buffer containing NP-40 (pH 6.0, Sigma) and heated in a microwave oven (600W) for 10 min. Endogenous peroxidase was blocked with 2% hydrogen peroxide in 70% methanol for 10 min at room temperature. Sections were incubated with primary antibodies for 2 hr, biotinylated secondary antibodies for 20 min at room temperature, and then subsequently processed by the avidin-biotin peroxidase complex method with 3-amino-9-ethylcarbazole (AEC) as the chromogen. Sections were lightly counterstained with Mayer's hematoxylin and viewed under a light microscope. Negative control sections were also incubated, but without primary antibody.

### Electrophoretic mobility shift assay for NF-κB and AP-1

Nuclear extracts were prepared as described [26]. Intestinal mucosa were harvested in hypotonic buffer and pelleted by centrifugation. The pellets were suspended in nuclear extract buffer. After 15 min on ice the suspensions were centrifuged and the supernatants were transferred to new tubes. The Bandshift kit (Promega Corp. Madison, WI) was used according to the manufacturer's instructions. Consensus and control oligonucleotides (Santa Cruz Biotechnology Inc.) were labeled by polynucleotides sequences included the AP-1 consensus (5' to 3') (CGCTTGATGACTTGGCCGGAA) or the NF-κB consensus(5' to 3') (AGTTGAGGGGAC-TTTCCCAGGC) (1.75pmol/μl). After the oligonucleotide was radiolabeled, 5 μg of nuclear protein was incubated with 2 μg of poly(dI-dC) and 5,000-10,000 cpm of γ[^32^P]-ATP-labeled oligonucleotides. After 30 min at room temperature, the samples were analyzed on a 4% polyacrylamide gel. The gel was dried and visualized by autoradiography.

### Statistical analysis

Values are expressed as means SEs. Intergroup comparisons were made using one-way ANOVA followed by Bonferroni correction. Statistical analysis was performed on Prism software (GraphPad). Data were expressed as mean ± standard deviation of the mean in all figures, and *p *< 0.05 is considered to be statistical significance. The bacterial count, MPO activity, FITC, glutathione level, and bacterial translocation between groups were assessed with one-way analysis of variance (ANOVA), followed by Scheffe's *F *test.

## Results

### Thermal injury induced intestinal permeability

**Figure 1:**
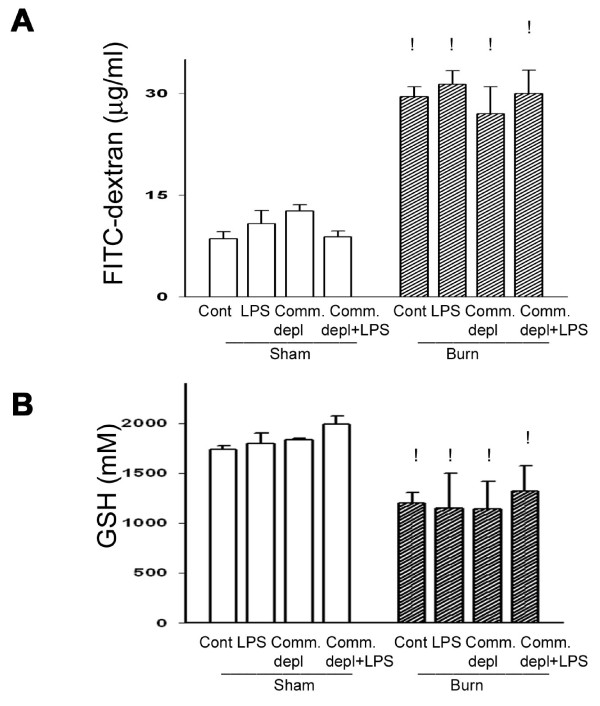
**Thermal injury induced permeability and decreased glutathione (GSH) level of intestinal mucosa**. (A) Thermal injury induced intestinal permeability and commensal depletion with or without LPS supplement did not change it. ! *p *< 0.05, vs. sham group. (B) Thermal injury decreased GSH level of intestinal mucosa and commensal depletion with or without LPS supplement did not change it. ! *p *< 0.05, vs. sham group.

To study the role of commensal microflora on thermal injury-induced intestinal dysfunction, we assessed the intestinal permeability of mice after oral antibiotics treatment with or without thermal injury. Thermal injury significantly increased intestinal permeability up to 356% of that of sham group at 24 hr after thermal injury (24.86 ± 3.56 vs. 8.56 ± 0.75 μg/ml). There was no significant difference of intestinal permeability between oral antibiotics group and control group. LPS supplement did not change intestinal permeability in mice after thermal injury compared with antibiotics + burn group or burn group (Figure 1A).

### Thermal injury decreased glutathione level of intestinal mucosa

Several reports indicated that GSH levels of various tissues including lungs, liver, kidney, and intestine were significantly decreased after thermal injury [27]. Thermal injury resulted in a significant decrease, by 26%, in glutathione level of intestinal mucosa compared with that of sham group (1307 ± 230 vs. 1777 ± 311 mM). Oral antibiotics treatment with or without LPS supplement did not change GSH level of intestinal mucosa compared with that of control group in burned animals (Figure 1B).

### TLR4 ligand decreased thermal injury-induced bacterial translocation

**Figure 2:**
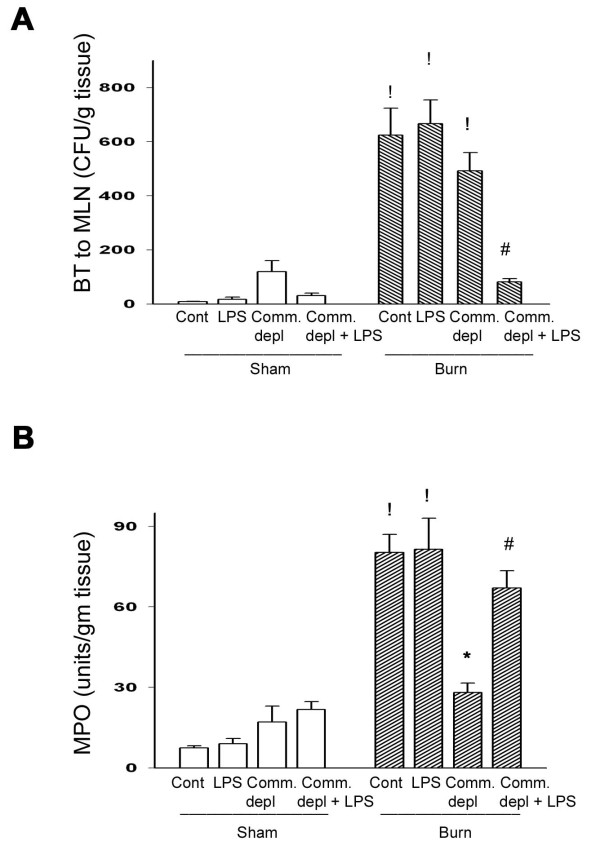
**TLR4 ligand decreased thermal injury-induced bacterial translocation and increased neutrophil accumulation in lung**. (A) LPS supplement in oral antibiotics decreased thermal injury-induced bacterial translocation in comparison with commensal depletion + burn group. ! *p *< 0.05, vs. sham group. ^# ^*p *< 0.05, vs. commensal depletion + burn group. (B) Commensal depletion significantly decreased thermal injury-induced lung myeloperoxidase (MPO) activity and LPS supplement reversed it. ! *p *< 0.05, vs. sham group. * *p *< 0.05, vs. burn group. ^# ^*p *< 0.05, vs. commensal depletion + burn group.

Four-week broad-spectrum antibiotics depleted bacteria in colonic fecal matter of mice as previously described [28]. Thermal injury significantly increased BT to MLNs in both control mice (from 8 to 620 CFU/g) and commensal depletion group (from 120 to 495 CFU/g tissue, about 8 fold increase). However, statistic analysis showed no significant difference of BT between commensal depletion group and burn group (495 ± 95 vs. 620 ± 172 CFU/g tissue). Most interestingly, LPS supplement in commensal depletion group significantly decreased 81% of thermal injury-induced BT when compared with that of commensal depletion + burn group (Figure 2A).

### TLR4 ligand increased thermal injury-induced neutrophil accumulation in lung

Lung MPO activity has been used as an index for pulmonary neutrophil accumulation [29]. Thermal injury induced a marked 11-fold increase of MPO activity in lung compared with control group (87 ± 8.17 vs. 6.73 ± 0.8 Units/g tissue) (Figure 2B). Oral antibiotics treatment with thermal injury induced a significant increase of MPO activity compared with commensal depletion group (28.09 ± 2.01 vs. 17.1 ± 4.2 Units/g tissue). However, Oral antibiotics treatment led to a significant 68% decrease (28.09 ± 4.97 vs. 87 ± 8.17 Units/g tissue) of thermal injury-induced lung MPO activity in comparison with that of burn group (Figure 2B). LPS supplement significantly increased 139% of MPO activity in lung compared with that of antibiotics + burn group. This demonstrates that commensal depletion decreases thermal injury-induced neutrophil deposition in lung and LPS supplement reverses it.

### TLR4 ligand increased TLR4 and TNFα expression in lung after burn

**Figure 3:**
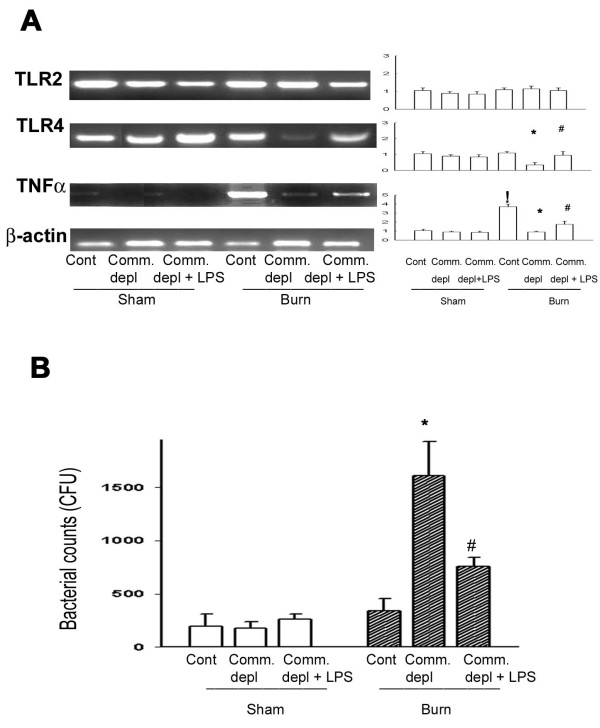
**TLR4 ligand increased TNFα expression in lung and the bacterial killing activity of peritoneal cells**. (A) Commensal depletion decreased TLR4 as well as TNFα mRNA expression in lung and LPS supplement reversed them. The values represent the average fold increase (± s.d.) in mRNA expression in 3 different mice of each group. ! *p *< 0.05, vs. sham group. * *p *< 0.05, vs. burn group. ^# ^*p *< 0.05, vs. commensal depletion + burn group. (B) Commensal depletion induced a significant increase of bacterial retention of peritoneal cells in burn mice compared with that of burn group. LPS supplement significantly decreased 55% of bacterial retention compared with that of antibiotics + burn group. * *p *< 0.05, vs. burn group. ^# ^*p *< 0.05, vs. commensal depletion + burn group.

To study the change of cytokines in lung in response to commensal depletion in mice, we examined TLR2, TLR4, and TNFα mRNA expression of lung in different groups. Oral antibiotics significantly decreased TLR4 and TNFα mRNA of lung in burn mice compared with those of burn group (Figure 3A). LPS supplement significantly increased TLR4 and TNFα mRNA expression of lung compared with those of antibiotics + burn group.

### TLR4 ligand increased bacterial killing activity of peritoneal cells in burn group

To define the effect of TLR4 ligand on the host defense to bacteria challenge, we harvested peritoneal cells from mice after oral antibiotics and examined the bacterial killing activity of cells. Peritoneal cells were cultured with E.coli and bacterial killing activity was determined by counting the E. coli remained. There was no significant difference in bacterial killing activity of peritoneal cells among control group, oral antibiotics group, and antibiotics + LPS group in sham burn animals (Figure 3B). However, antibiotics with burn treatment induced a significant 10-fold increase of bacterial retention compared with that of burn group (1732 ± 410, vs. 290 ± 65 CFU). LPS supplement significantly decreased 55% of bacterial retention compared with that of antibiotics + burn group. These results indicate that commensal depletion decreases the bacterial killing activity of peritoneal cells in burn and LPS supplement reverses it.

### TLR4 ligand increased TLR4 expression of intestinal mucosa

**Figure 4:**
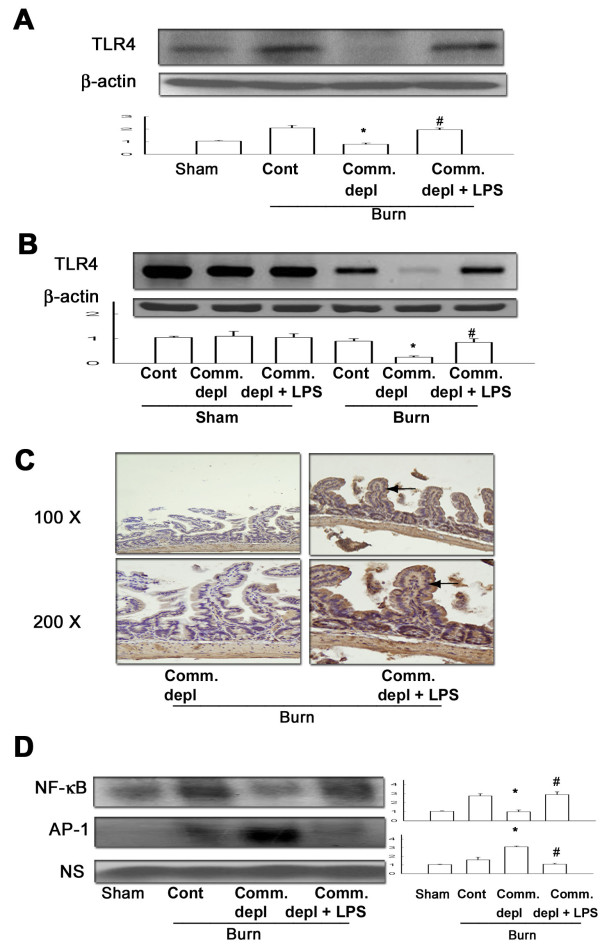
**TLR4 ligand increased TLR4 expression and NF-κB DNA binding activity of intestinal mucosa after thermal injury**. (A) LPS supplement increased TLR4 protein expression of intestinal mucosa in commensal depleted mice. * *p *< 0.05, vs. burn group. ^# ^*p *< 0.05, vs. commensal depletion + burn group. (B) LPS supplement increased TLR4 mRNA expression of intestinal mucosa in commensal depleted mice. The values represent the average fold increase (± s.d.) in 3 different mice of each group. * *p *< 0.05, vs. burn group. ^# ^*p *< 0.05, vs. commensal depletion + burn group. (C) LPS supplement increased TLR4 immunostaining of intestinal epithelial cells. (D) Commensal depletion decreased the NF-κB DNA-binding activity of intestinal mucosa and LPS supplement increased it. (*n *= 4). *p < 0.05, vs. burn group. * *p *< 0.05, vs. commensal depletion + burn group. NS = non-specific binding.

Oral antibiotics treatment significantly decreased TLR4 protein (Figure 4A) and mRNA (Figure 4B) expression of intestinal mucosa in burn mice compared with burn only group. LPS supplement significantly increased TLR4 protein and mRNA expression of intestinal mucosa in thermal injured mice in comparison with commensal depletion + burn group.

### TLR4 ligand increased TLR4 expression of intestinal epithelial cells

To examine which cells in intestine expressed toll-like receptors after oral LPS supplement, we evaluated the TLR4 protein expression in intestine with immunohistochemical staining. We found that LPS supplement in oral antibiotics significantly increased the TLR4 expression of intestinal epithelial cells in burn group when compared with antibiotics + burn group (Figure 4C).

### TLR4 ligand increased NF-κB but decreases AP-1 activity of intestinal mucosa after thermal injury

NF-***κ***B is an integrator of different signals involved in inflammatory responses in the gut [30]. Commensal depletion decreased NF-***κ***B but increased AP-1 DNA-binding activity of intestinal mucosa in thermal injured mice compared with those of burn group. LPS supplement increased NF-***κ***B but decreased AP-1 DNA-binding activity of intestinal mucosa in burn mice compared with those of commensal depletion + burn group (Figure 4D).

### Oral TLR4 ligand supplement after burn decreased bacterial translocation

**Figure 5:**
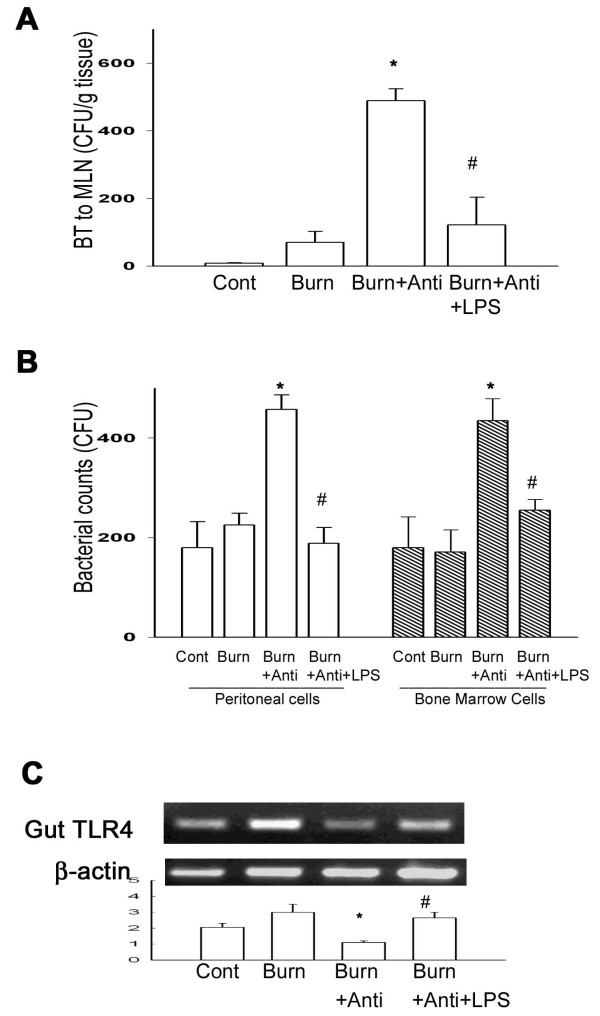
**Oral TLR4 ligand supplement after burn decreased bacterial translocation (BT) and increased bacterial killing activity of macrophages as well as TLR4 expression of intestinal mucosa**. (A) Oral antibiotics feeding after burn (Burn+Anti) increased BT to MLN in comparison to burn group and LPS supplement reversed it. * *p *< 0.05, vs. burn group. ^# ^*p *< 0.05, vs. burn + antibiotics group. (B) Oral antibiotics feeding after burn induced a significant increase of bacterial retention in both peritoneal macrophages and bone marrow cells. LPS supplement (Burn+Anti+LPS) decreased bacterial retention of peritoneal macrophages and bone marrow cells in comparison with antibiotics + burn group (Burn + Anti). * *p *< 0.05, vs. burn group. ^# ^*p *< 0.05, vs. antibiotics + burn group. (C) Oral antibiotics feeding after burn decreased TLR4 mRNA expression of intestinal mucosa in comparison with burn group and LPS supplement increased it. * *p *< 0.05, vs. burn group. ^# ^*p *< 0.05, vs. burn + antibiotics group

Thermal injury induced an increase of BT to MLNs at 48 hr after thermal injury (70 ± 35 CFU/g tissue). Oral antibiotics feeding after burn significantly increased BT to MLN in comparison with burn group (489 ± 61 CFU/g tissue, about 6 fold increase). Whereas, LPS supplement in drinking water markedly decreased 75% of BT in comparison with that of burn + antibiotics group (Figure 5A).

### Oral TLR4 ligand supplement after burn increased bacterial killing activity of peritoneal cells and bone marrow cells

To simulate the clinical burn condition, we gave WT mice oral antibiotics with or without LPS supplement after burn and examined the bacterial killing activity of peritoneal cells and bone marrow cells at 48 hr after thermal injury. Thermal injury with saline feeding did not change the bacterial killing activity of peritoneal macrophages or bone marrow cells in comparison with control group (Figure 5B). However, oral antibiotics administration after burn significantly increased 116% and 221% of bacterial retention in peritoneal macrophages and bone marrow cells, respectively, in comparison with that of burn group. LPS supplement significantly decreased 61% and 41% of bacterial retention in peritoneal macrophages and bone marrow cells, respectively, in comparison with that of antibiotics + burn group.

### Oral TLR4 ligand supplement after burn increased TLR4 mRNA expression of intestinal mucosa

We examined the TLR4 mRNA expression of intestinal mucosa in different groups. Oral antibiotics feeding after burn significantly decreased TLR4 mRNA expression of intestinal mucosa compared with that of burn group (Figure 5C). LPS supplement significantly increased TLR4 mRNA expression of intestinal mucosa compared with that of antibiotics + burn group.

### TLR4 ligand did not change bacterial translocation in C3H/HeJ mice

**Figure 6:**
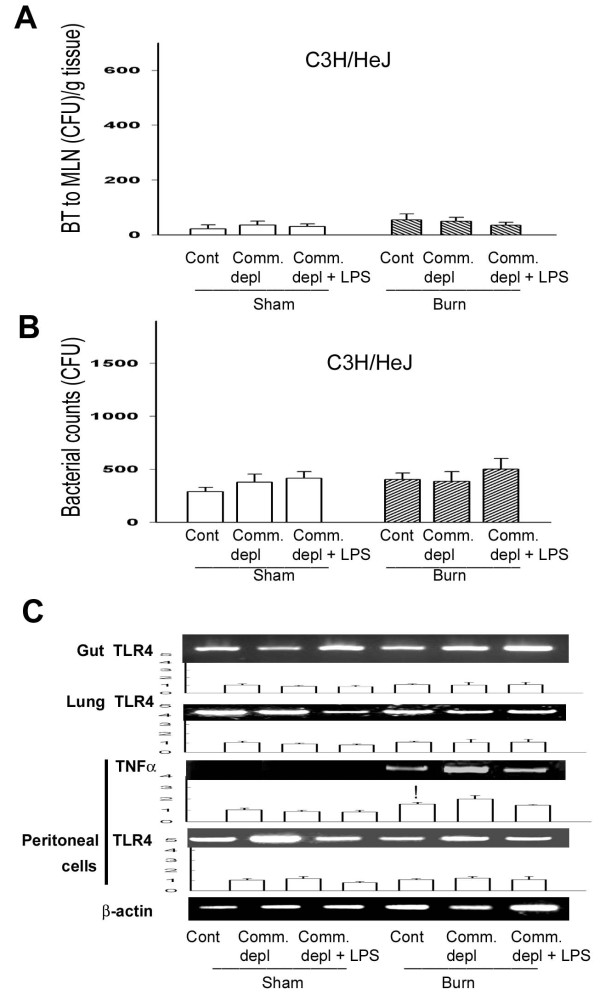
**TLR4 ligand did not change bacterial translocation (BT), bacterial killing activity of peritoneal cells, and TNFα mRNA expression in C3H/HeJ mice**. (A) Commensal depletion with or without LPS supplement did not change BT in C3H/HeJ mice. (B) Commensal depletion with or without LPS supplement did not change bacterial retention of peritoneal cells in C3H/HeJ mice. (C) Commensal depletion with or without LPS supplement did not change TNFα mRNA expression of peritoneal cells in C3H/HeJ mice. The values represent the average fold increase (± s.d.) in mRNA expression in 3 different mice of each group. ! *p *< 0.05, vs. sham group.

To further define the involvement of TLR4 on the change of bacterial translocation to MLNs after thermal injury, we examined BT to MLNs in C3H/HeJ mice after thermal injury with oral antibiotics treatment. Thermal injury did not induce bacterial translocation to MLNs in control group and commensal depleted group in C3H/HeJ mice. Commensal depletion with burn did on change BT to MLNs in C3H/HeJ mice when compared with burn group. Furthermore, LPS supplement did not change BT when compared with commensal depletion group in C3H/HeJ mice (Figure 6A).

### TLR4 ligand did not change the bacterial killing activity of peritoneal cells in C3H/HeJ mice

To further define the role of TLR4 on host defense against bacterial challenge after commensal depletion, we examined the bacterial killing activity of peritoneal cells in C3H/HeJ mice after different treatments. Commensal depletion did not change the bacterial killing activity of peritoneal cells in C3H/HeJ mice when compared with burn group (Figure 6B). Furthermore, LPS supplement did not change bacterial killing activity of peritoneal cells in C3H/HeJ mice. There is no significant difference of peritoneal cell count between WT mice and C3H/HeJ mice (Table 1).

**Table 1:** Components of peritoneal cells of C3H/HeJ and wild-type (WT) mice.

Group	Monos (%)	Lymphos (%)	PMN (%)	Macrophages (%)	Mast cells (%)
WT	51.7 ± 7.3	42.6 ± 4.1	2.9 ± 0.5	1.9 ± 0.3	0.9 ± 0.1
C3H/HeJ	54 ± 8	39.6 ± 5.1	3.2 ± 0.9	2.1 ± 0.5	1.1 ± 0.3

### TLR4 ligand did not change TLR4 and TNFα expression in C3H/HEJ mice

To further evaluate the involvement of TLR4 in the cytokine change of different tissues after commensal depletion, we examined TLR4 mRNA expression of gut, lung, and peritoneal cells in C3H/HeJ mice. Thermal injury induced TNFα mRNA expression of peritoneal cells in C3H/HeJ mice when compared with that of sham burn group. Commensal depletion with or without LPS supplement did not change TLR4 mRNA expression of gut, lung, and peritoneal cells in C3H/HeJ mice when compared with control group. Also, commensal depletion with or without LPS supplement did not change TNFα mRNA expression of peritoneal cells in C3H/HeJ mice when compared with burn group (Figure 6C).

## Discussion

Major burn in humans and experimental animals is associated with compromised immune functions and increased acute gastrointestinal derangement that disrupts gastrointestinal mucosa integrity and facilitates the bacterial translocation. Our data demonstrate that commensal microflora are critical in maintaining innate immunity in burn through TLR4 signaling and oral TLR4 ligand supplement reverses the commensal depletion effect in burn through the decrease of BT and the increase of bacterial killing activity.

Provision of enteral nutrients shortly after injury alters gut flora and protects the immuno-compromised, stressed, or thermally injured patients through unknown mechanism [8]. The cross talk between commensal microflora in intestinal tract and systemic innate immunity is still undefined. Previous paper demonstrated that recognition of commensal microflora by toll-like receptors was required for intestinal homeostasis [13]. Here, our results show that commensal microflora in intestinal tract induce TLR4 expression of intestinal epithelial cells and enhance the bacterial killing activity of inflammatory cells in burn. Previously, macrophage of TLR4-deficient mice demonstrated impaired bacterial recognition and phagocytosis compared with wild-type mice [31]. Our present data show that commensal depletion does not change the bacterial killing activity and TLRα mRNA expression of peritoneal cells in C3H/HeJ mice. Altogether, our data suggest that TLR4 signaling in intestinal tract is important in inducing bacterial killing activity of inflammatory cells after thermal injury and commensal microflora are critical in maintaining innate immunity in burn through TLR4 signaling. Previous paper has proved that enteral nutrients shortly after injury could alter intestinal flora [8]. Accordingly, the beneficiary effect of early enteral nutrients on systemic immunity in burn could be through the reestablishment of gut flora in intestinal tract. In a recent prospective randomized controlled trial of using probiotics in pancreatitis patients, patients who received the probiotics had a surprisingly high rate of nonocclusive bowel necrosis [32]. This paper used 10 billion probiotic bacteria per day on top of enteral nutrition. This might have even further increased local oxygen demand, with a combined deleterious effect on an already critically reduced blood flow. On the contrary, we provide animals combined antibiotics to deplete commensal microflora and stimulate the TLR4 expression in intestinal mucosa with oral LPS supplement. All in all, our data imply that oral supplement of certain flora to activate TLR4 in intestinal mucosa but not overload the intestinal flora could be a new therapeutic strategy to enhance systemic innate immunity in major burn patients.

The second important conclusion to be derived from the present results is that TLR4 ligand supplement decreases burn-induced BT and reverses commensal depletion-induced reduction of bacterial killing activity in burn. First, we demonstrate that thermal injury induces a significant increase of bacterial translocation to mesenteric lymph nodes. LPS supplement stimulates the TLR4 expression of intestinal mucosa and significantly decreases thermal injury-induced bacterial translocation. Thermal injury is associated with mesenteric vasoconstriction that lead to damage of the gut mucosa and dysfunction of the gut barrier, resulting in an increased gut permeability, and absorption of bacteria and bacterial toxins [33]. Burn-induced gut injury results in the production of biologically active factors that are carried in the mesenteric lymph, but not the portal plasma, which injure endothelial cells and activate neutrophil and contribute to distant organ injury [15]. The potential cause of sepsis and subsequent multiple organ failure following thermal injury could be the failure of intestinal mucosa to act as barrier against BT [34]. BT is a phenomenon in which live bacteria or its products cross the intestinal barrier. The function of the gut barrier depends on mucous epithelia (mechanical barrier) and secreting IgA and immune cells (immune barrier) [35]. Gut translocation of bacteria has been shown in both animal and human studies. BT and its complications have been shown clearly to occur in animal models, but its existence and importance in humans has been difficult to ascertain [35]. Our results demonstrate that TLR4 ligand significantly increases the bacterial killing activity of inflammatory cells and decreases thermal injury-induced bacterial translocation but has no effect on intestinal permeability or glutathione level. These results suggest that TLR4 ligand improves the thermal injury-induced gut barrier function through the enhancement of the intestinal innate immunity (immune barrier) rather than the decrease of intestinal permeability (mechanical barrier). Second, LPS supplement reverses commensal depletion-induced reduction of bacterial killing activity of peritoneal cells after burn. Previously, TLRs of those macrophages which were resident in the lamina propria of the intestine has been implicated closely related to the potent inflammatory response, intestinal inflammation, and corresponding injury [36]. On the contrary, our data demonstrate that LPS supplement reverses commensal depletion-induced reduction of bacterial killing activity of peritoneal cells in WT mice but not in C3H/HeJ mice. This indicates that LPS supplement induces host defense to bacterial challenge in burn through the TLR4 signaling. Recent research indicates that there is significant phylogenetic and diversity in TLR4-mediated responses [37]. A new TLR4 ligand that is originated from LPS structures but without its toxicity could be developed in the future to enhance host defense in major burn patients.

Infection is a common source of morbidity and mortality in critically burned patients. Burn injury produces a complex interaction of gut-derived inflammatory reaction paralleled by systemic inflammatory responses. The use of selective decontamination of the digestive tract (SDD) is of particular interest for application in patients with major burn injury where the need for intubation and mechanical ventilation likely contributes to oropharyngeal and intestinal colonization with pathogenic microorganisms. Recently, SDD treatment of experimental burn in adult rats resulted in attenuated septic challenge-related inflammatory responses and improved myocardial contractile response [5]. However, our data demonstrate that oral antibiotics treatment after burn is not without a cost: decreasing bacterial killing activity of inflammatory cells. Also, our results suggest that oral TLR4 ligand supplement could reverse the unfavorable effect of oral antibiotics treatment in burn through the increase of gut barrier function and bacterial killing activity of inflammatory cells.

TLRs are membrane signaling receptors that play essential roles in innate defense against microbes. TLRs are type I integral membrane glycoproteins that contain leucine-rich repeats glanced by characteristic cysteine-rich motifs in their extracellular regions and a cytoplasmic TIR homology domain. Ligand-induced TLR dimerization permits the binding of cytoplasmic adapter proteins, MyD88, to the TLR cytoplasmic tails [38]. A major downstream effect of TLR signaling is the activation of the transcription factor NF-κB, which is required for expression of many genes related to innate immunity and inflammation [12]. Studies have proved that TLR4 stimulation maintained intestinal hemostasis through the NF-κB activation of the intestinal mucosa [13]. The stimulatory effect of LPS supplement on NF-κB activation of intestinal mucosa further corroborates that LPS supplement enhances host defense in burn through the increase of TLR4 signaling. Activation of the NF-κB transcription factor pathways is an essential immediate early step of immune activation [39]. Altogether, our data suggest that TLR4 ligand activates the NF-κB activation in intestinal mucosa and enhances the gut barrier function after thermal injury.

Septic shock has been reported to be the most common cause of death in the noncoronary intensive care unit [40]. Pulmonary sepsis is the septic complication most frequently encountered in severely burn patients [41]. MPO system plays an important role in the microbicidal activity of phagocytes and neutrophil play an essential role in the Human's innate immune response to infection [42]. MPO, released by neutrophil, may attack normal tissue and thus contribute to the pathogenesis disease. Our data demonstrate that commensal depletion significantly decreases TLR4 as well as TNFα expression in lung and LPS supplement increases TLR4 as well as TNFα expression in lung after burn. NF-κB family members control transcriptional activity of various promoters of proinflammatory cytokines, cell surface receptors, transcription factors, and adhesion molecules that are involved in intestinal inflammation such as TNFα[12]. Our data suggest that TLR4 ligand stimulation in the gut enhances TLR4 expression and neutrophil deposition in lung. The stimulatory effect of LPS supplement on TNFα expression as well as MPO activity of lung could be through the induction of NF-κB activation.

## Conclusions

In summary, commensal microflora induce gut barrier function and the bacterial killing activity of peritoneal cells in burn through the increase of toll-like receptor 4. TLR4 ligand reverses oral antibiotics effect in burn through the decrease of BT and the increase of bacterial killing activity.

## Abbreviations

TLRs: toll-like receptors; MLNs: mesenteric lymph nodes; LPS: lipopolysaccharide; BT: bacterial translocation; LTA: lipoteichoic acid; TBSA: total body surface area; FITC: fluorescein isothiocyanate; MPO: myeloperoxidase; WT: Wild type; GSH: glutathione

## Competing interests

The authors declare that they have no competing interests.

## Authors' contributions

LWC and CMH designed research; WJC and PHC performed research; LWC and CMH analyzed data; LWC and CMH wrote the paper. All authors read and approved the final manuscript.
